# Mechanical and Morphological Effect of Plant Based Antimicrobial Solutions on Maxillofacial Silicone Elastomer

**DOI:** 10.3390/ma11060925

**Published:** 2018-05-30

**Authors:** Sophia Tetteh, Richard J. Bibb, Simon J. Martin

**Affiliations:** 1 Loughborough Design School, Loughborough University, Loughborough, Leicestershire LE11 3TU, UK; r.j.bibb@lboro.ac.uk; 2 Materials, Loughborough University, Loughborough, Leicestershire LE11 3TU, UK; s.j.martin@lboro.ac.uk

**Keywords:** antimicrobial solution, mechanical testing, facial prostheses, tensile strength

## Abstract

The objective of this study was to determine the effect of plant based antimicrobial solutions specifically tea tree and Manuka oil on facial silicone elastomers. The purpose of this in vitro study was to evaluate the effect of disinfection with plant extract solution on mechanical properties and morphology on the silicone elastomer. Test specimens were subjected to disinfection using tea tree oil, Manuka oil and the *staphylococcus epidermidis* bacteria. Furthermore, a procedure duration was used in the disinfection process to simulate up to one year of usage. Over 500 test specimens were fabricated for all tests performed namely hardness, elongation, tensile, tear strength tests, visual inspection and lastly surface characterization using SEM. A repeated measures ANOVA revealed that hardness and elongation at break varied significantly over the time period, whereas this was not observed in the tear and tensile strength parameters of the test samples.

## 1. Introduction

Maxillofacial prostheses are usually fabricated to mask facial defects or deformities in patients, especially when surgery is not feasible. These defects or deformities are usually caused by cancer, traumatic accidents or congenital diseases and may affect speech, quality of speech, quality of life, psychology and social behavior. Deterioration of the prostheses is usually caused by environmental exposure namely ultraviolet (UV) light, air pollution and changes in humidity and temperature; and by handling, cleaning and removal of the prosthesis [1,2,3,4,5,6,7]. Research shows that these prostheses generally last 13 to 28 months [8,9,10].

Some desirable mechanical properties of maxillofacial prostheses are hardness; tensile strength, tear strength, elongation and many others. The hardness of maxillofacial material is a measure of its flexibility and should be comparable to that of the anatomic facial tissue [11,12]. Tensile strength is important in order to express overall strength of a material while hardness indicates the strength of a materials surface [11]. Elongation is a measure of flexibility and an indicator of the overall flexibility of a prostheses material with facial prostheses elastomer to rupture during use and maintenance [11]. Hence, it accommodates facial movement [12,13,14].

### 1.1. Degradation by Environment

The effect of weather on polymeric materials has strong dependence on geographic location, season, time of day, cloud cover and exposure orientation, since the critical weather factors vary with these conditions [15]. Environmental characteristics that cause degradation are sunlight, temperature, moisture, wind, dust and pollutants [15,16]. Weathering of polymers can induce changes in physical and chemical characteristics that result in significant modifications to their appearance, mechanical properties and thermal properties [15,16]. Polymer deterioration due to weathering is a result of a photo-oxidative attack, namely a combined action of oxygen and sunlight on a material’s chemical structure [16]. The photo-oxidative degradation causes an initial formation of free radicals; reaction of free radicals with oxygen, production of polymer oxy- and peroxy-radicals and secondary polymer radicals resulting in chain scission. Finally, a reaction of different free radicals with each other results in crosslinking [15] as stated elsewhere. The changes in physical properties affect the polymer’s structural network, namely the density, which causes an increase in crosslinking due to the formation of bonds between the existing monomers or between the chains.

### 1.2. Degradation by Cleaning/Handling

The prostheses can be exposed to mucosa, moist air and skin secretions (such as sweat, sebum and other products secreted by the skin), subsequently leading to a multilayer biofilm formation [9,17,18]. Biofilms are a structured community of cells enclosed in a self-produced polymeric matrix adherent to an inert or living surface [19]. Contamination of the prostheses’ surface can harbor microorganisms within its pores if not removed by cleaning [9,20]. For facial prostheses, it is unknown what percentage of failure is related to microorganisms, but adhesion of microorganisms to and formation of biofilms on surfaces of prostheses are well known causes for infections of medical devices [21]. Problems associated with microbial colonization of these prostheses are black stains on the prostheses surface, offensive odors and tissue infections [9].

Chronic infections by biofilms are of interest because of resistance of microorganisms present in the biofilm to antibiotics. Furthermore, the biofilm architecture, i.e., the layer on the surface of a prosthesis in which the cells are embedded in extracellular polysaccharide matrix, results in poor penetration of antibiotics through that layer [22]. Furthermore, nutritional support either extrinsically or intrinsically from within the prostheses will sustain further growth of these micro-organisms [17]. Disinfection of elastomers could alter their surface characteristics and the bulk of the material due to the extraction of some compounds from the matrix to disinfection solutions or the water as indicated in many studies [23,24]. Current cleaning methods for facial prostheses require patients to clean their prostheses meticulously. These cleaning methods include: Using a cotton bud soaked with mild soapy solution [17]; using a soft nylon bristled toothbrush and a mild detergent soap [20,25]; rinsing in tap water, pat dried with a towel and stored in a container away from direct light or heat or exposed to cigarette smoke [25]. Cleansing, care and storage conditions post-fabrication make the prostheses vulnerable to bacterial and fungal growth. Furthermore, surface topography (i.e., the roughness, surface irregularities, etc.), the hydrophobicity and chemistry all have an influence on the attachment of microorganisms to a surface. Neutral soap; sodium hypochlorite 1%; cleansing tablets; and chlorhexidine are some of the most commonly used cleansing agents for the facial silicone elastomer [7,26].

Furthermore, a study to elucidate the degree of satisfaction after maxillofacial prosthetic rehabilitation revealed that clinical efforts to ensure all patients were cognizant of the care of their prostheses [27]. Nevertheless, there was a misunderstanding relating to lack of soap and water usage; usage of inappropriate solvents; and infrequent cleansing that hastens the deterioration of prosthetic polymers, especially the periphery of the prostheses to the importance of keeping the prostheses meticulously clean [27].

Hence, it is essential to concentrate on the efficacy of the differing cleaning methods and materials to help establish suitable cleaning methods and or agents not only for peri-implant tissues but also for the maxillofacial prosthesis. Research has shown that frequent exposure to chemical disinfection may interfere with the properties of the silicone [23,28,29,30,31], hence it is essential to find alternative methods of disinfection. Furthermore, increased resistance of bacteria to antibiotics, adverse effects such as toxicity, allergenic and even mutagenic effects of some antibacterial agents currently used in dentistry and financial constraints [32] make it vital to explore other options of disinfection. Natural phytochemicals from phytotherapy is one possible option worth exploring as has been shown to be safe, effective and economical [32].

Natural antimicrobials are obtained from different sources, namely plants, animals, bacteria, algae and fungi [33]. Usage of broad spectrum antibiotics increases the apparent resistance to almost all commercially available antimicrobial drugs [34]. Antimicrobial compounds from plants serve as a defence mechanism against predation by microorganisms, insects, and herbivores either through their odors, or flavor [35]. The antimicrobial phytochemicals can be divided into several categories namely phenolics; terpenoids; alkaloids; lectins and polypeptides and polyacetylenes [35].

Antimicrobials utilized in this study (tea tree and Manuka oil) were chosen based on documented and/or anecdotal antimicrobial effects. Furthermore, a thorough search revealed that these oils had not previously been used as a cleanser for maxillofacial prostheses.

Tea tree oil is incorporated as the principal or natural preservative in many pharmaceutical and cosmetic products intended for external use [36]. It is derived from the Australian native plant *Melaleuca alternifolia* [37,38] and produced by steam distillation of its leaves and terminal branches [37,39]. Once condensed, the clear pale yellow oil is separated from the aqueous distillate [37]. The antimicrobial activity of *Melaleuca* may be mediated by interaction with and disruption of bacterial plasma membranes [36], whilst the anti-inflammatory activity may be attributed by the suppression of reactive oxygen species in monocytes and neutrophils [39,40].

Manuka (*Leptospernum scoparium*) is a bushy shrub that has deep green fragrant leaves that bears small white to pink flowers distributed throughout New Zealand in widely varying climates, altitudes and population densities [41]. The tree sheds its bark in long papery strips and has been traditionally used to treat many ailments [42]. It is touted as an antibacterial, antiseptic, antifungal and anti-inflammatory agent [43]. The active constituent of Manuka oil is thought to be beta-triketone [44,45].

The purpose of this study was to evaluate the mechanical and morphological behavior alterations of the maxillofacial prosthetic elastomer during longevity studies due to plant-based antimicrobial solutions and contaminated with *staphylococcus epidermidis*. The plant-based antimicrobial solutions from extensive search, and, to the best of the knowledge of the authors, have not previously been used as a cleansing agent for maxillofacial prostheses material. The null hypothesis was that the usage of plant-based antimicrobial solution in a longevity study would not affect the mechanical properties (elongation, tensile strength, tear strength and Shore A hardness) of the maxillofacial silicone elastomer.

## 2. Results

### 2.1. Mechanical Testing

**Figure 1:**
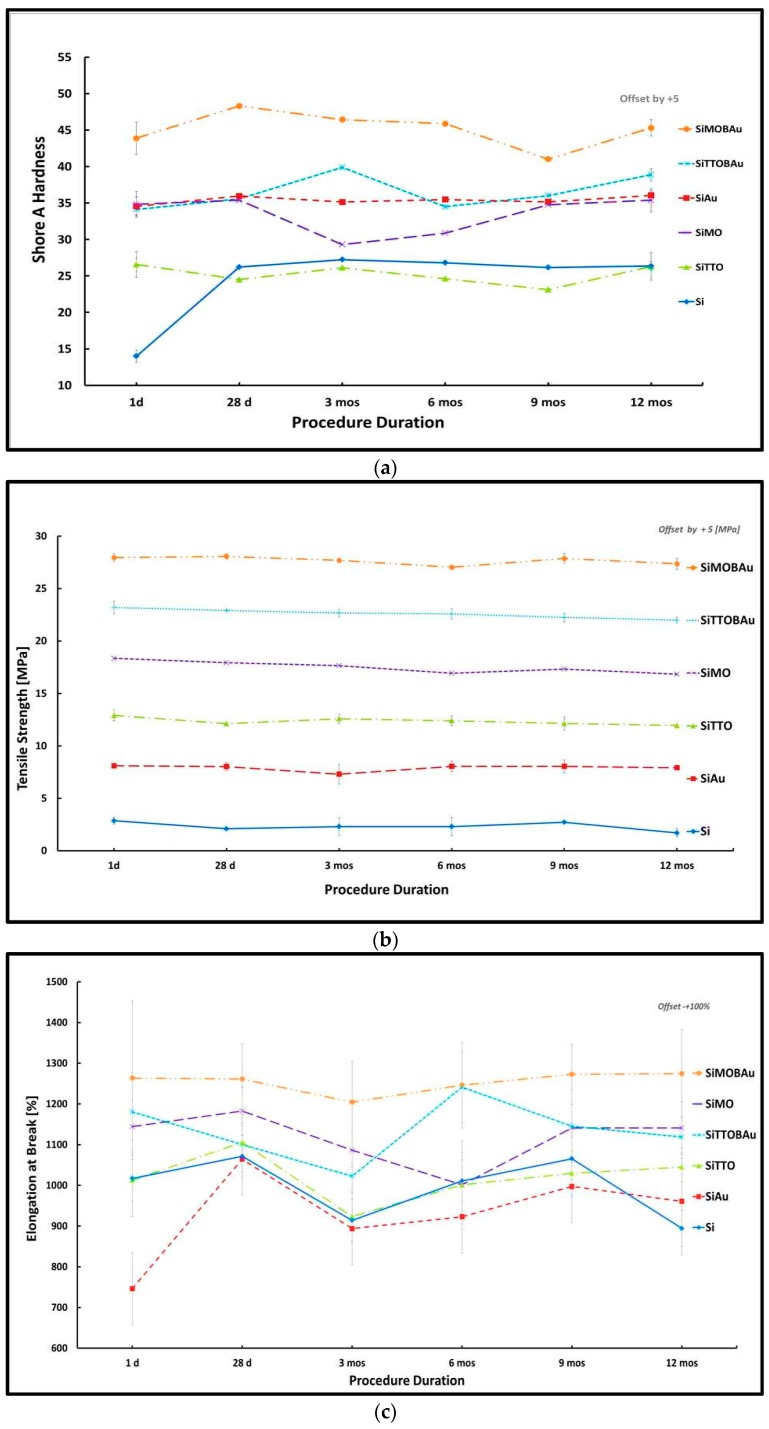

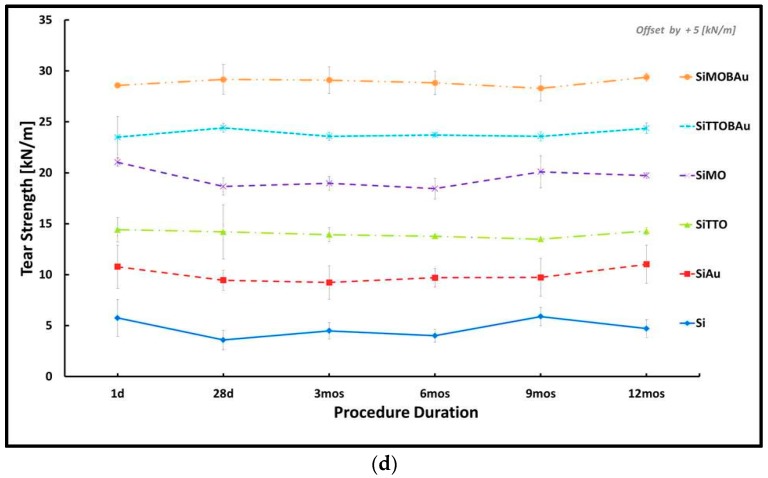
(**a**) line plot displaying means; standard deviation error bars and offset (+5—SiAu; +10—SiTTO; +15—SiMO; +20—SiTTOBAu; +25—SiMOBAu) for Shore A hardness samples. (**b**) line plot displaying means; standard deviation error bars and offset (+5 MPa—SiAu; +10 MPa —SiTTO; +15 MPa —SiMO; +20 MPa —SiTTOBAu; +25 MPa —SiMOBAu) for tensile strength samples; (**c**) line plot displaying means; standard deviation error bars and offset (+100%—SiAu; +200%—SiTTO; +300%—SiMO; +400%—SiTTOBAu; +500%—SiMOBAu) for elongation at break samples; (**d**) line plots displaying means; standard deviation error bars and offset (+5 kN/m —SiAu; +10 kN/m —SiTTO; +15 kN/m —SiMO; +20 kN/m —SiTTOBAu; +25 kN/m —SiMOBAu) for tear strength samples.

A summary of the line plots of the mechanical parameters with standard deviation (SD) depicted by error bars are shown in Figure 1a–d.

Regression Analyses: Furthermore, a linear regression analysis for each of the mechanical parameters with their respective predictors at the specific time periods. Table 1 reports adjusted *R*^2^, *R*^2^ and F statistics from the regression analyses results run. The results from the regression analysis for the mechanical parameters of hardness, elongation and tear strength revealed that the varying time periods variables were statistically significant as summarized F (6, 41) = 10.082, *p* < 0.0005, *R*^2^ = 0.537; F (6, 10) = 8.876, *p* < 0.002, *R*^2^ = 0.842 and F (6, 10) = 3.459, *p* < 0.002, *R*^2^ = 0.675, respectively. However, regression results from tensile strength was not statistically significant across the varying time periods; F (6, 10) = 2.457, *p* > 0.524, *R*^2^ = 0.596.

ANOVA Analyses: The null hypothesis was tested using a mixed design ANOVA repeated measures, where the independent variables were the conditioned silicone samples and test conditions (procedure time). The dependent variables were the Shore A hardness and tensile properties (tear strength, elongation percentage at break; tensile strength). The independent variables were the procedural times. Significant differences detected by the ANOVA (*p* < 0.05) prompted pairwise comparisons with Bonferroni *post hoc* tests.

In comparing the effect of the conditioned samples with time periods, we conducted a six (conditioned sample: Silicone (control sample) (Si); silicone autoclaved (SiAu); silicone with tea tree oil (SiTTO); silicone with Manuka oil (SiMO); silicone with tea tree oil, *staphylococcus epidermidis* and autoclaved (SiTTOBAu) and silicone with Manuka oil, *staphylococcus epidermidis* and autoclaved (SiMOBAu)) ×10 (Time: 24 h, 28 days, 3 months, 6 months, 9 months, 12 months) repeated measures ANOVA.

Mauchly’s test of sphericity, which determines the differences between conditions are equal indicated that the assumption of sphericity had been violated for the within subjects’ effect χ^2^ (14) = 25.605, *p* = 0.034 (elongation); χ^2^ (14) = 74.746, *p* < 0.05 (hardness). Therefore, the degrees of freedom were corrected using Greenhouse-Geisser estimates ε = 0.483 (elongation) and ε = 0.504 for the main effect of hardness tensile strength respectively. Meanwhile, tensile and tear strength parameter did not indicate any violation of the sphericity condition effect χ^2^ (14) = 19.320, *p* = 0.166 and χ^2^ (14) = 13.897, *p* = 0.474, respectively.

Furthermore, the mixed design ANOVA repeated measures with a Greenhouse-Geisser correction as seen in Table 2. Furthermore, the mixed design ANOVA repeated measures with a Greenhouse-Geisser correction for the Shore A hardness and elongation of the samples were statistically significantly different thus (F (2.893, 121.517) = 12.720, *p* < 0.0005, *R*^2^ = 0.537, *η*^2^ = 0.232) and (F (2.414, 26.559) = 3.909, *p* = 0.026, *R*^2^ = 0.842, *η*^2^ = 0.262), respectively. Hence, the results suggest that there was a statistical significance on the main effects of Shore A hardness and elongation at break with the procedure times.

The interaction of the procedure time with the hardness samples was statistically significant (F (25, 121.517) = 11.080, *p* < 0.0001, *η*^2^ = 0.569), indicating the effect of the procedure time on the hardness of the samples differed. *Post hoc* tests using Bonferroni correction revealed a statistically significant reduction by an average of 1.723 (*p* < 0.003) between day 1 and month 1 (*p* < 0.003); 2.098 (*p* < 0.0001) between day 1 and 12 months, 1.298 (*p* < 0.0001) between month 6 and 1 months, 1.502 (*p* < 0.001) between month 1 and 9 months, 1.169 (*p* = 0.014) between month 9 and 3 months and then reduced additionally by 1.877 between month 9 to 12 (*p* < 0.0001).

The interaction of the procedure time with the elongation samples were not statistically significant F (12.072, 26.559) = 1.187, *p* = 0.292, *η*^2^ = 0.350), indicating that the main effect of the procedure time on the elongation of the test sample did not differ over the procedure time. Bonferroni *post hoc* tests revealed statistically significant differences of a mean increment of 113.211 (month 1 to 3; *p* < 0.009); 103.557 (month 1 and 12 months; *p* < 0.027) and further reduced by-86.118 (month 9 to 12; *p* < 0.015).

Additionally, for the tensile strength of samples, the difference between the means were significantly different (F (5, 55) = 10.093, *p* < 0.0005, *η*^2^ = 0.478), respectively. A Bonferroni *post hoc* test revealed a significant difference in the means of the tensile strength samples 0.607 (day 1 and 3 month; *p* < 0.10); 0.756 (day 1 and month 4; *p* < 0.010); 0.996 (day 1 and 12 months; *p* < 0.0001), one month and 12 months an increase of 0.592 (*p* < 0.007), month 9 and 12 a reduction in −0.756 (*p* < 0.10). However, the interaction effect between the tensile strength samples and the procedure time (F (25, 55) = 1.593, *p* = 0.076, *η*^2^ = 0.420) revealed non-statistical significance. *Post hoc* Bonferroni results disclosed a statistical significance between procedure time 1 month and samples (Si and SiMOBAu) mean reduction of 0.978 (*p* < 0.039); month 12 and sample (Si and SiAu) mean reduction of 1.244 (*p* < 0.010); month 12 and sample (SiAu and SiTTO, SiAu and SiMO, respectively) mean increment of 0.980 (*p* < 0.047) and 1.099 (*p* < 0.021), respectively.

Additionally, for tear strength, the difference between the means was not statistically significant different at (F (5, 25) = 1.049, *p* = 0.399, *η*^2^ = 0.087), respectively. Interaction effects of the procedure time with tear strength (F (25, 55) = 0.601, *p* = 0.917, *η*^2^ = 0.215) were also not statistically significant.

Furthermore, examination into the conditioned silicone hardness and tensile strength samples showed that the control Si and SiAu hardness samples differed significantly over the varying time periods, which could be due to the high temperature of the autoclaving process influencing the crosslinking of the SiAu hardness test samples. In addition, the SiTTO and SiMO hardness samples were also significantly different, even though the antimicrobial solutions utilised in the study were from the same family, whilst the tensile strength samples were significantly the same. Likewise, the SiMOBAu and SiTTOBAu hardness and tensile strength samples were significantly the same, which indicates the presence of the *staphylococcus epidermidis* and the usage of the antimicrobial solutions caused the same effect in the samples, though initially with the SiTTO and SiMO hardness samples they differed significantly over the time period.

### 2.2. Morphological Testing

Visual observations revealed a brownish yellowish colour for the SiTTO; SiMO, SiTTOBAu and SiMOBAu samples. This indicates that the antimicrobial solutions tea tree and Manuka; the presence of antimicrobial compounds; or even the autoclaving process was having an effect on the test samples.

**Figure 2:**
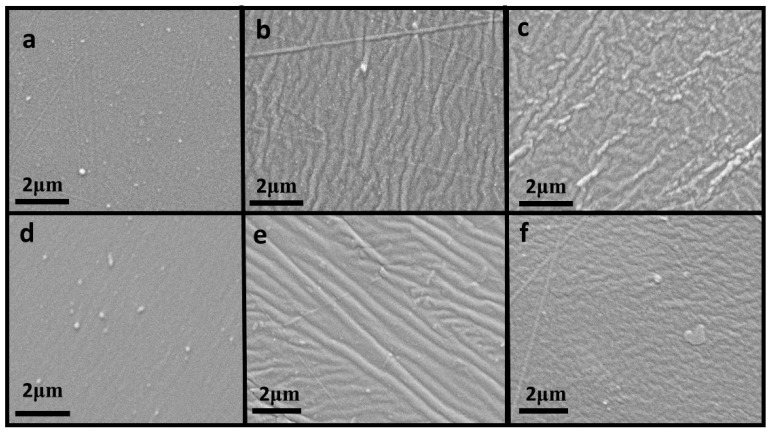
Photomicrograph of test samples at magnification of 5 K. (**a**) SiTTOBAu at Day 1 (**b**) SiTTOBAu at 6 months (**c**) SiTTOBAu at 12 months (**d**) SiMOBAu at Day 1 (**e**) SiMOBAu at 6 months and (**f**) SiMOBAu at 12 months.

Silicone elastomer test samples were examined by SEM after the visual observation for any visible changes. Using a 120, 1 K, 5 K and 10 K revealed deterioration of the of the prostheses material for the procedure duration. The higher magnification revealed the extent of deterioration of the prosthesis material. Figure 2 displays changes over the day 1; 6-month and 12-month periods at a magnification of 5 K.

## 3. Discussion

For the mechanical parameters hardness and elongation, we accept the hypothesis that variance differences over the time period were significant. However, for tear and tensile strength, we reject the null hypothesis that, at the varying time periods, were not significantly different.

Overall, the general pattern of the hardness of the samples increases slightly within the 1- to 28-day period and then levels out across all test samples up until month 9, then slightly increases until month 12. Furthermore, the tensile strength of the samples decreases for all samples from day 1 to month 6 and then increases slightly from month 6 to month 9 and further decreases slightly until month 12. Additionally, the elongation at break of the samples increases from day 1 to day 28 and then decreases from 28 days to three months and then increases between months 3 to 6 and then commences to decrease across all samples. In addition, the tear strength of the samples decreases sharply from day 1 to 28 days and then almost levels out from 28 days to six months and then gradually starts to increase between months 6 to 9 and then increases for all samples except for Si samples, which decreases. Overall, as the hardness of the samples increases over the procedure duration, the tensile strength seemed to decrease whilst the elongation at break and tear strength revealed no definite pattern over that same procedure duration.

Overall, statistically significant differences were observed in hardness test samples as well as the procedure duration. This may have resulted from the resistance of the silicone samples to compression during the testing.

Accelerated aging has been shown to have an effect on the aging of silicone properties, mostly because photo-oxidation and hydrolysis of silicone are the main degradation reactions and they occur after the material has been exposed to sunlight, air, humidity and temperature [26]. Similar studies have attributed an increment or decrement of hardness to surface alterations and not water absorption [30]; the decomposition of the cleaning solution into carbon monoxide, carbon dioxide and sulfur dioxide, which could lead to either a hardening or a softening of materials [46]. Similarly, an increment and decrement in hardness of silicone after disinfection has been attributed to continuous polymerization of the silicone; the polymerization process can usually be slowed by a reaction between the disinfection products, absorption of the disinfection solution that could lead to a porous structure [47]. Another study [48] indicated significant differences in hardness, percentage elongation, tensile strength and tear strength in three maxillofacial silicone materials (M511, Z004 and M511) with thixotropic agents added. The low molecular weight polymer chains of M511 are considered the weakest points in the cross-linked structure, hence leading to low tensile and tear strength [48]. However, a low cross-link density between polymer chains allows more elasticity in tension and reduces rigidity and hardness [48], which might explain the results obtained in the current study.

Photomicrographs from this study indicated that over time the surface of the maxillofacial prostheses developed ridges, which could be due to the antimicrobial solutions as well as dead bacteria on the surface of the test samples. The ridges observed on the surface could in effect serve as a source in which bacteria grow. 

Only one type of maxillofacial silicone material and only a single type of bacteria (*staphylococcus epidermidis*) were utilized for this study. Additionally, other well-known factors such as sweat, mucosa, saliva and many others that have been found to cause biofilm formation can be incorporated into future research design. Furthermore, maxillofacial prosthesis materials from different manufacturers or a variety of different bacteria might cause the results observed in this study to vary. Hence, proprietary information of the components of the silicone from the different manufacturers makes it arduous to assess the transferability of the results obtained. Furthermore, studies in determining the effects of other fungi or bacteria such as *Candida* spp., *Enterobacter cloacae*, *Staphylococcus aureus*, *Serratia marcesens*, *Pseudomonas aeruginosa* and many others might need to be performed in the future to gain better insight of its effect on prosthesis materials. In addition, surface analyses such as abrasive wear, surface roughness, micro-indentation and many others need to be performed to prove the extent and rate of degradation of the surface of the maxillofacial material. Additionally, longitudinal color change and conditional factors such as water intake and porosity can also be evaluated to fully understand the degradation process.

## 4. Materials and Methods

All raw materials; manufacturers and batch/lot numbers utilized in this research are listed in Table 3. The mechanical properties, Shore A hardness, tensile strength, percentage elongation at break and tear strength were investigated with antimicrobial solution and disinfected with *staphylococcus epidermidis*.

### 4.1. Sample Preparation

#### 4.1.1. Silicone Elastomer

Maxillofacial silicone elastomer was mixed manually for 10 min in a ratio of 10:1 (base: catalyst) according to the manufacturer’s instructions. The silicone elastomer utilized in this study uses a cross-linking system via an addition reaction. The silicone base contains vinyl terminated polydimethylsiloxanes, surface treated silica fillers and a Platinum complex as catalysts [48]. The cross-linker (part B) contained a hydrogen siloxane polymer. The silicone was then poured into polycarbonate plates and its surface was smoothened until it was level throughout the plate. Air bubble elimination was achieved by using a pressure pot or storing in a refrigerator to slow the curing process. The samples were then put into an oven at 70 °C for 60 to 80 min. After polymerization, the silicone was taken out of the polycarbonate plates and stored. Test samples were then cut out from these silicone sheets. All test specimens were visually inspected to ensure that they were free of any defects and specimens with air bubbles were discarded.

#### 4.1.2. Antimicrobial Disinfectant

The *staphylococcus epidermidis* was utilized for this study, since it is part of the human skin flora as well as its availability and accessibility.

#### 4.1.3. Bacterial Preparation

Nutrient broth and agar solutions were prepared following the manufacturer’s instruction. The nutrient agar and broth solutions were then autoclaved (Astell Scientific Portaclave, Size 4) prior to usage at 121 °C for 15 min. After autoclaving, the nutrient agar was stored in a water bath at 60 °C, whilst the nutrient broth is inoculated with a loop full of *staphylococcus epidermidis*. This solution was then left in the incubator at 37 °C overnight (24 h) for the growth of the *staphylococcus epidermidis* before usage. Bacterial cultures of *staphylococcus epidermidis* were made from stock cultures of American Type Culture Collection ATCC 14990/NCIMB 12721 (Manassas, VA, USA). These were grown in nutrient broth (Oxoid CM001), inoculated and incubated at 37 °C overnight. Nutrient agar (LABM) plates were then prepared and seeded with 1 mL of the overnight culture.

#### 4.1.4. Antimicrobial Disinfectant Minimum Inhibitory Concentration

Antimicrobials utilized in this study (tea tree and Manuka oil) were chosen based on documented and/or anecdotal antimicrobial effects. Furthermore, a thorough search revealed that these oils had not previously been used as a cleanser for maxillofacial prostheses.

Varying concentrations of both antimicrobial solutions were prepared in an increasing two-fold dilution series concentration volumes as shown in Table 4 in determining the minimum inhibitory concentration over 24, 48 and 72 h, respectively, in an infected *staphylococcus epidermidis* agar plate. The minimum mean concentration volumes at which a distinct clear zone is observed was noted. The concentration volume of antimicrobial solutions (tear tree and Manuka oil) that inhibited the growth of the *staphylococcus epidermidis* bacteria cultures are shown in Table 5.

#### 4.1.5. Conditioning of Samples for Mechanical Testing

Standardized cut test pieces were conditioned for mechanical testing. The conditioning applied are as follows: a. silicone (control sample) (Si); b. silicone autoclaved (Si/Au); c. silicone with Tea tree oil (SiTTO); d. silicone with Manuka oil (SiMO); e. silicone with tea tree oil, *staphylococcus epidermidis* and autoclaved (SiTTOBAu) and f. silicone with Manuka oil, *staphylococcus epidermidis* and autoclaved (SiMOBAu). Conditioning with tea tree and Manuka oil (SiTTO and SiMO) was done by placing the silicone test samples in a petri dish filled with distilled water and the minimum inhibitory concentration of the antimicrobial solution, while conditioning of (SiTTOBAu) and (SiMOBAu) was done with the test samples put in a solution of *staphylococcus epidermidis* for 24 h and afterwards placed in a petri dish filled with distilled water and the minimum inhibitory concentration of the antimicrobial solutions.

#### 4.1.6. Conditioning Time Periods with Antimicrobial Solution

The simulated times utilized were 1 day; 28 days; 3 months; 6 months; 9 months and 12 months, respectively. Simulated times from 1 day to 28 days were followed accordingly whilst simulated times commencing from 3 months to 12 months followed a procedure duration as similarly performed elsewhere [30]. Procedure duration was adopted to reduce the risk of any infection or contamination. All conditioned samples infected with *staphylococcus epidermidis* were autoclaved after contact with antimicrobial solutions. The procedure duration time series utilised is shown in Table 6.

### 4.2. Mechanical (Quantitative) Testing

#### 4.2.1. Hardness

Hardness specimens (25 mm by 25 mm by 6 mm) were cut manually into the test samples. Three hardness specimens were prepared for each test criteria. The specimens were tested according to ASTM D 2240 [49]. A Shore A durometer (Durometer Type M, Shore Instrument and Mfg Co., Inc., New York, NY, USA) was utilised. For each specimen, eight (8) readings were taken with 6 mm distance maintained between readings and of the edges of test specimen.

#### 4.2.2. Tensile Strength

Three type 2 dumbbell shaped tensile tests specimens were fabricated as per ISO specification number 37, type 1 [50]. Tensile grips were installed on a universal testing machine (Lloyd LR50K Tensometer Test Machine, Lloyds Instruments Ltd., West Sussex, UK) with a separation of 20 mm between them. The tensile strength tests were performed at 50 mm/min crosshead speed with a 50 N load cell. Prior to testing, the thickness of each specimen was measured using a thickness gauge at the centre and the end of each test specimen. The average thicknesses were then used for cross-sectional area calculations. The tensile strength (Ts) and elongation percentage at break (EP) is then calculated automatically using Equations (1) and (2):

(1) $$\mathrm{Ts}=\frac{F_{b}}{w_{t}},$$

(2) $$\mathrm{EP}=\frac{L_{b}-L_{o}}{L_{o}}\times 100,$$

where F_b_ is the force recorded at break (N), w_t_ is the width of the narrow portion of the specimen (mm), t is the thickness of the test length, L_o_ is initial test length and L_b_ is the test length at break (mm).

#### 4.2.3. Tear Strength Test

Trouser shaped tear strength test specimens were fabricated 100 mm × 15 mm × 2 mm and tested according to the ISO 34-1 using a (Lloyd LR50K Tensometer Test Machine, Lloyds Instruments Ltd., West Sussex, UK) [51]. A cut in the test specimen was made at the centre of the width of the test piece (40 mm long). The test specimen trouser legs were inserted symmetrically and in axial alignment with the direction of the pull in each grip. The depth of insertion was 30 mm ensuring the specimens was held adequately firmly. Specimens were tested at a strain rate of 100 mm/min. The tear strength is expressed in kN/m and calculated by Equation (3), where Ts is tear strength, F is force at break and d is the thickness of the test sample:

(3) $$\mathrm{Ts}=\frac{F}{d}.$$

### 4.3. Morphology Testing

#### 4.3.1. Visual Observation

Visual observation was observed for each of the samples after the conditioning as well as after testing of the samples. The observations were noted and recorded.

#### 4.3.2. Scanning Electron Microscopy Testing

The scanning electron microscope (SEM) instrument images the surface of the sample by scanning it with a high-energy beam of electrons. The SEM was used to examine any structural and changes on the surface of the test samples.

#### 4.3.3. Characterization of Test Samples

Scanning electron microscopic (SEM) examination was performed using an analytical scanning electron microscope (FIB–SEM Nova 600 NanoLab, FEI Company, Hillsboro, OR, USA) to monitor any changes within the surface of the silicone elastomer matrix as well as within due to microbial infection and the usage of the plant based disinfectants.

#### 4.3.4. Sample Preparation for Scanning

Thin cross sections of the silicone sample were prepared and mounted rigidly on specimen holders. The silicone elastomer is non-conductive, hence the specimens were coated with an ultrathin coating of gold, by sputtering and the images were taken using a low voltage SEM at 150 kV.

### 4.4. Data Analysis

Descriptive statistics were generated from all quantitative data specifically means; standard deviations and variances using SPSS for windows software (IBM SPSS 24.0, Inc., Chicago, IL, USA). Furthermore, data from the quantitative studies of the experimental groups were collected and compared to the control group using a repeated measure analysis of variance (ANOVA) for tensile strength, percentage elongation at break, tear strength and Shore A hardness [52] as well as a regression analysis.

For qualitative data obtained, visual colour changes observed during the conditioning and testing are noted. Images obtained from the SEM are also observed to note any changes in the structure and surface of the samples under testing.

## 5. Conclusions

Within the limitations of this study, Shore A hardness and elongation properties revealed changes after disinfection as compared to tear and tensile strength mechanical properties, which did not. In addition, surface changes and colour within the test samples revealed that perhaps microbial infections as well as disinfection have an effect on the maxillofacial silicone during the long-term period.

## Figures and Tables

**Table 1:** Regression summary of mechanical parameters.

Mechanical Parameters	*R*	*R* ^2^	Adjusted *R* Square	F	*df* ^2^	Sig. F Change
Hardness	0.772	0.596	0.537	10.082	41	0.000
Tensile	0.772	0.596	0.353	2.457	10	0.100
Tear Strength	0.821	0.675	0.480	3.459	10	0.041
Elongation	0.918	0.842	0.747	8.876	10	0.002

*df* = degree of freedom.

**Table 2:** ANOVA table for mechanical parameters in this study.

Mechanical Parameters	Source	*df*	*SS*	*MS*	F	*p*
Hardness	Time (within)	2.893	183.105	63.287	12.72	0.000
	Time × Silicone (within)	14.466	797.509	55.129	11.080	0.000
Tensile	Time (within)	5	9.484	1.897	10.093	0.000
	Time × Silicone (within)	25	7.484	0.299	1.593	0.076
Elongation at break	Time (within)	1.00	249,062.363	249,062.363	3.909	0.074
	Time × Silicone (within)	12.072	378,233.869	31,330.260	1.187	0.341
Tear Strength	Time (within)	5	7,620,067.608	1,524,012.521	1.049	0.389
	Time × Silicone (within)	25	21,850,848.090	874,033.924	0.601	0.917

*df* = degree of freedom; *SS* = sum of squares; *MS* = mean square.

**Table 3:** Materials utilized in this research study.

Materials	Manufacturer	Batch/Lot Number
M511 Platinum Silicone Part A	Technovent, Bridgend, Wales, UK	B17D/B17AH
M511 Platinum Silicone Part B	Technovent, Bridgend, Wales, UK	B16C/B17D
Manuka Oil	Essential Oils Direct, Oldham, UK	8583/9124
Tea Tree Oil	Essential Oils Direct, Oldham, UK	9100

**Table 4:** Varying concentrations of antimicrobial solutions.

Volume Used	Volume/Volume Percent Solution (*v*/*v*) %									
	%	0.05	0.1	0.2	0.4	1.0	2.0	4.0	8.0	16.0
**Tea Tree**	µL	15	30	60	120	300	600	1200	-	-
**Manuka**		-	-	2	4	10	20	40	80	160

**Table 5:** Varying concentrations of antimicrobial solutions.

Test Agent	*Staphylococcus Epidermidis*
Tea Tree Oil	0.2% (*v*/*v*)
Manuka Oil	0.4% (*v*/*v*)

**Table 6:** Simulated time periods utilised for conditioning test samples.

Procedure Duration Utilised for Conditioning Samples						
Simulated Time (m—months; d—days)	12 m	9 m	6 m	3 m	28 d	1 d
Procedure Time (hours)	30	22.5	15	7.5	2.5	0.083

## Abbreviations

ANOVA	Analysis of variance
ATCC	American Type Culture Collection
Fb	the force recorded at break
°C	degrees Celsius
SD	Standard deviation
SEM	Scanning electron microscopy
Si	silicone (control sample)
SiAu	silicone autoclaved
SiMO	silicone with Manuka oil
SiMOBAu	silicone with manuka oil, *staphylococcus epidermidis* and autoclaved
SiTTO	silicone with tea tree oil
SiTTOBAu	silicone with tea tree oil, *staphylococcus epidermidis* and autoclaved
UV	Ultraviolet
